# Effects of Calcium Silicate-Based Sealer Residues on Adhesive Bonding to Coronal Dentin: An in Vitro Study

**DOI:** 10.34172/joddd.44162

**Published:** 2026-03-30

**Authors:** Mariana Bena Gelio, Thais Piragine Leandrin, Ana Lídia Pinheiro Silva Sato, Milton Carlos Kuga, Joissi Ferrari Zaniboni

**Affiliations:** ^1^Department of Restorative Dentistry, Araraquara School of Dentistry, São Paulo State University - UNESP, Araraquara, SP, Brazil; ^2^Department of Restorative Dentistry, School of Dentistry of Ribeirão Preto, University of São Paulo, Ribeirão Preto, SP, Brazil

**Keywords:** Bond strength, Calcium silicate, Dentin, Endodontics, Ethanol, Sealer

## Abstract

**Introduction:**

This study aimed to evaluate the influence of residues from three endodontic sealers on the adhesive interface of two universal adhesive systems: Scotchbond Universal (SU) and OptiBond All-in-One (OU).

**Methods:**

Forty bovine dentin surfaces were cleaned with 95% ethanol and divided into 4 groups: control, without endodontic sealer; AH, dentin impregnated with AH PLUS sealer; ES, EndoSequence BC Sealer; and MTA, MTA Fillapex. After cleaning, the persistence of residues and the number of open dentinal tubules were analyzed by scanning electron microscopy. Sixty bovine incisor crowns were divided into six groups (n=10): AH-SU, ES-SU, MTA-SU, AH-OU, ES-OU, and MTA-OU to evaluate the microshear bond strength in a universal testing machine and failure mode, in stereomicroscopy. Data were analyzed using the Kruskal-Wallis test, ANOVA, and post hoc Tukey tests (α=0.05).

**Results:**

AH Plus showed higher persistence of residues and fewer open tubules compared to other groups (*P*<0.05), whereas EndoSequence BC Sealer and MTA Fillapex did not differ from each other (*P*>0.05). Bond strength was lower in AH Plus groups, regardless of the adhesive system used (*P*<0.05). EndoSequence BC Sealer and MTA Fillapex exhibited similar and higher bond strength than AH Plus (*P*>0.05). Adhesive failure was the most frequent mode in all groups, except for MTA-SU, which showed a higher incidence of mixed failures.

**Conclusion:**

Residues of endodontic sealers affect dentinal penetrability and the adhesion of universal adhesives. In the present study, EndoSequence BC Sealer and MTA Fillapex had less impact on the adhesive interface than AH Plus, suggesting a lower impact on adhesion after cleaning with 95% ethanol.

## Graphical Abstract

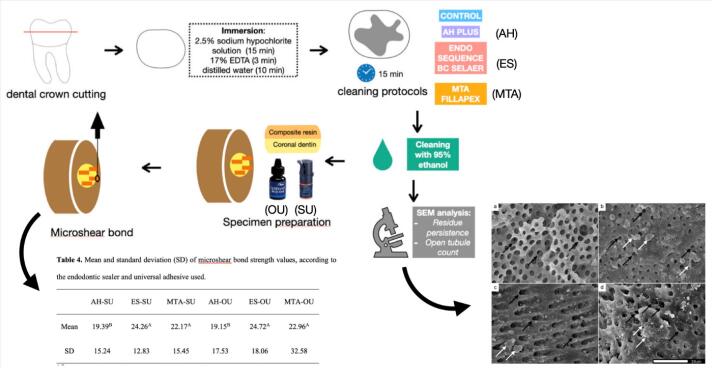

## Introduction

 The success of endodontic treatment depends not only on effective disinfection and obturation of the root canal system but also on the proper selection of endodontic sealers. These materials should exhibit biocompatibility, ease of handling and insertion, and physicochemical properties that ensure a durable seal between the dentinal walls and gutta-percha cones.^1,2^ Endodontic sealers can be classified according to their chemical composition into several categories: epoxy resin-based sealers, silicone-based sealers, and calcium silicate-based sealers, commonly referred to as bioceramics, which may incorporate Mineral Trioxide Aggregate (MTA) in their formulation.^1,3,4^

 Epoxy resin-based sealers, such as AH Plus, are widely used in clinical practice due to their excellent physicochemical stability and satisfactory biological performance.^4,5^ In contrast, MTA, introduced to dentistry in the 1990s,^1,3^ and subsequently calcium silicate-based sealers, have gained popularity in recent years due to their high biocompatibility, bioactivity, and ability to chemically interact with dentin. This interaction occurs through the release of calcium ions and subsequent calcium phosphate precipitation, leading to the formation of hydroxyapatite.^2,4,6^

 However, despite these favorable properties, residual sealer may remain on dentinal walls after obturation, particularly in the coronal region. These residues can compromise the bonding of restorative materials. In the case of bioceramic sealers, calcium ion release promotes apatite formation within dentinal tubules, which may alter the dentin substrate and interfere with adhesive penetration.^7,8^ Consequently, the long-term success of adhesive restorations in endodontically treated teeth is closely related to the integrity of the adhesive interface, which can be significantly affected by the chemical composition of the sealer used.^9^

 To minimize the adverse effects of persistent residues, several cleaning protocols have been proposed. Ethanol, in particular, has been widely employed as an effective organic solvent, capable of cleaning the dentin surface and removing resinous and oily residues, especially for epoxy resin-based sealers.^10,11^ Calcium silicate-based sealers are hydrophilic and set in the presence of moisture; their ionic and mineral-based matrices may be more easily disrupted by water or polar solvents such as ethanol, facilitating residue removal.^12^

 Universal adhesives have gained increasing popularity due to their versatility and simplified application protocols, allowing their use in both etch-and-rinse and self-etch strategies.^13^ Among their functional monomers, 10-methacryloyloxydecyl dihydrogen phosphate (MDP) and glycerophosphate dimethacrylate (GPDM) are the most commonly employed, both capable of forming stable chemical bonds with hydroxyapatite, contributing to improved bond strength.^14^ However, the behavior of these adhesives when applied to dentin surfaces contaminated with residues from calcium silicate-based sealer remains unclear.

 Previous investigations have demonstrated that residual endodontic sealers can adversely affect adhesion to coronal dentin by reducing dentinal tubule patency, hindering monomer infiltration, and compromising hybrid layer formation. Studies evaluating epoxy resin-based sealers,^15,16^ such as AH Plus, have reported significant difficulties in eliminating residues, resulting in decreased bonding performance even when different cleaning protocols were applied. Roberts et al.^2^ reported that AH Plus contamination significantly reduced bonding effectiveness, even after various removal protocols, whereas bioceramic materials showed less aggressive interference. Other studies have also demonstrated that calcium silicate-based sealers promote ionic exchange and hydroxyapatite deposition, which may modify the dentin substrate and influence the interaction with functional monomers in universal adhesives.^17-19^

 Therefore, this study aimed to evaluate the influence of persistence of residues from three different endodontic sealers on the adhesive interface of two universal adhesive systems: Scotchbond Universal and OptiBond All-in-One. After cleaning the dentin with 95% ethanol, the following parameters were assessed: persistence of residues, bond strength, and failure mode at the adhesive interface. The null hypotheses tested were: (I) No differences would be observed in the persistence of residues and open dentinal tubules among the different sealers; (II) No significant differences would be found in microshear bond strength between the different combinations of sealers and adhesive systems.

## Methods

 One hundred bovine incisor crowns were examined under × 20 magnification using a stereomicroscope (Leica Microsystems, Wetzlar, Germany) to ensure the absence of structural defects, such as cracks or fractures. Selected specimens were stored in a thymol solution at 4°C until further use. In accordance with Normative Resolution No. 30/2016 of the Brazilian National Council for the Control of Animal Experimentation (CONCEA), ethical approval by an Animal Research Ethics Committee was not required. Table 1 lists the materials used in this study.

**Table 1:** Materials, manufacturers, and chemical compositions of the materials used

**Materials**	**Manufacturer**	**Chemical Composition**
AH Plus	Dentsply De Trey, Konstanz, Germany	Paste A: Bisfenol-A resin; Bisfenol-F resin; calcium tungstate, zirconium oxide, sílica, iron oxide; Paste B: adamantine amine, N-dibenzil-5-oxanonane-diamine-1.9, TCD-diamine, calcium tungstate, zirconium oxide, silica, and silicon oil.
EndoSequence BC Sealer	Brasseler, Savannah, GA, USA	Zirconium oxide, calcium silicates, calcium phosphate monobasic, calcium hydroxide, filler, and thickening agents.
MTA Fillapex	Angelus, Londrina, PR, PR	Base Paste: salicilate resin, calcium tungstate, sílica nanoparticulates, pigments; Catalist Paste: diluente resin, mineral trioxide aggregate, sílica nanoparticulaes, pigments.
Scotchbond Universal	3M ESPE, St Paul, MN, USA	2-hydroxyethyl methacrylate: 15-25% Bisphenol A diglycidyl ether dimethacrylate (Bis GMA): 15-25% Decamethylene dimethacrylate: 5-15% Ethanol: 10-15% Sinalo-treated silica: 5-15% Water: 10-15% 1,10-Decanediol phosphate methacrylate Acrylic itaconic acid copolymer Camphorquinone N,N-DImethylbenzocaine: < 2
OptiBond All in One	Kerr, Brea, CA, USA	Acetone: 35‒45% Hydroxy Ethyl Meth Acrylate (HEMA): 8‒11% Ethyl alcohol: 4‒9% Disodium Hezafluorosilicate: 0.5‒1.5% Camphoroquinine Filler: Nano fillers

###  Persistence of Residues and Open Dentinal Tubules

**Figure 1:**
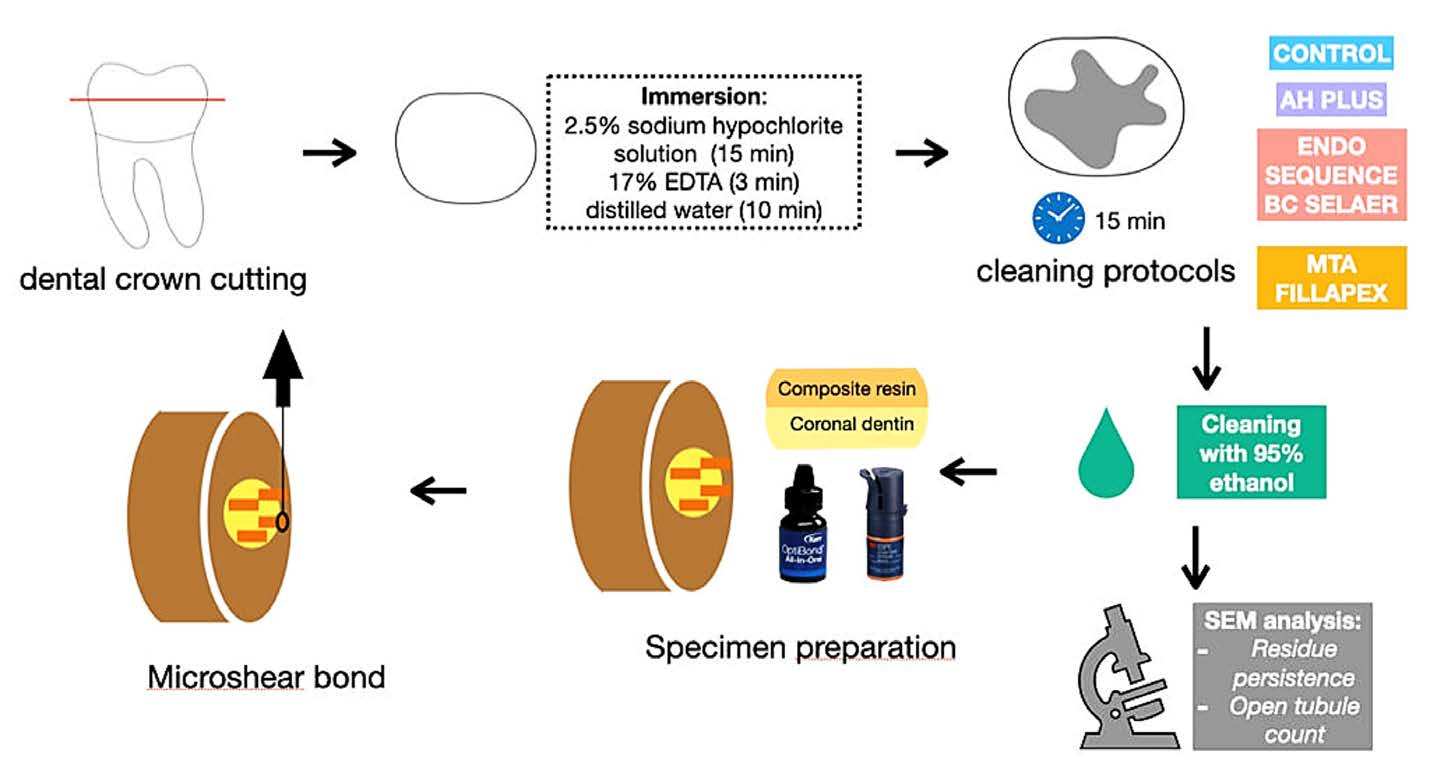
Scheme of the methodology

 Forty dentin specimens (5 × 10 mm) were obtained from longitudinal sections on the mesial and distal surfaces and, subsequently, transversely in the middle third of the tooth crown, using a low-speed diamond disc (Isomet; Buehler Ltd, Lake Bluff, IL, USA), under constant cooling with distilled water. The lingual surface was also removed to expose only the pulp chamber dentin.^20^ The specimens were immersed in a 2.5% sodium hypochlorite (Asfer, São Caetano do Sul, SP, BR) for 10 minutes, followed by immersion in 17% EDTA (Biomicina, Ibiporã, PR, BR) for 3 minutes.^20^ Subsequently, the specimens were rinsed with 10 mL of distilled water and dried with absorbent paper points (Dentsply, Pirassununga, SP, BR). Figure 1 shows the experimental design.

 The specimens were randomly assigned to four groups (n = 10), according to the endodontic sealer applied to the dentin surface:

 CON (negative control): The dentin surface was cleaned by rubbing a cotton pellet soaked in 95% ethanol (Rinse-n-Dry; Vista Dental, Racine, WI, USA).

 AH (AH Plus; Dentsply De Trey, Konstanz, Germany): The sealer was mixed at a 1:1 ratio as recommended by the manufacturer and applied uniformly to the dentin surface using a microbrush (KG Sorensen, Sao Paulo, SP, Brazil), forming a visible layer that remained for 15 minutes. Then, the surface was cleaned with a cotton pellet soaked in 95% ethanol (Rinse-N-Dry; Vista Dental, Racine, WI, USA) until no visible residue remained.

 ES (EndoSequence BC Sealer; Brasseler, Savannah, GA, USA): The dentin surface was moistened with distilled water, and the automix bioceramic sealer was directly applied to the surface. Application and removal of endodontic sealer were similar to those described in the AH group.

 MTA (MTA Fillapex; Angelus, Londrina, PR, BR): The sealer was prepared by mixing pastes A and B in a 1:1 ratio, following the manufacturer’s instructions. Application and removal followed the same steps as in the AH group.

 Then, the specimens were stored at room temperature for 7 days and dehydrated in a closed chamber containing silica gel for 24 hours. The specimens were mounted on metallic stubs, metallized with gold-palladium alloy (single cycle of 120 s) under vacuum, in a metallization chamber (MED 010, Balzers Union, Balzers, Liechtenstein) and examined under a scanning electron microscope (JEOL 6060; JEOL Ltd., Tokyo, JPN), operating at 15 kV. Four images were obtained from each specimen at × 500 magnification, and the most representative image was selected for analysis of residue persistence. Images were classified into scores according to the incidence of residues on the dentin surface, using a classification adapted from Kuga et al. (9): 0: absence of residues; 1: < 25% of the surface covered; 2: < 50% of the surface covered; 3: < 75% of the surface covered and 4: nearly the entire surface covered. Two independent, calibrated examiners (kappa = 0.82) evaluated the images.

 Additionally, the number of open dentinal tubules was quantified using ImageJ (NHI, USA), with only fully visible, unobstructed tubules counted as open.

###  Microshear Bond Strength and Failure Mode

 Sixty bovine incisor crowns were used in this test. The sample size was determined based on previous studies^21,22^ and a pilot study in which three specimens per group were tested. Using the means and standard deviations obtained from the pilot study, the required sample size was calculated to detect statistically significant differences between groups using two-way ANOVA, with a significance level of 5% and a statistical power of 80% (G*Power ver. 3.1 for Mac, Heinrich Heine University, Düsseldorf, Germany). Based on this calculation, ten specimens per group were selected.

 The buccal enamel surface of each crown was abraded using #80 silicon carbide sandpaper on a polisher machine (DP-10 Panambra; Struers, Ballerup, DK) until the underlying dentin was exposed and flattened, resulting in a standardized 10 × 10 mm dentin area. The dentin surface was protected with PVC film (Wyda, São Paulo, SP, BR), and each specimen was embedded in acrylic resin within a PVC cylinder (16.0 cm high × 2.54 cm wide), keeping the dentin surface exposed. After 24 hours, the exposed dentin was polished with 320- and 600-grit silicon carbide sandpaper at 500 rpm under constant water irrigation. Surface flatness and standardization were verified under a stereomicroscope (Model SZX7, Olympus, São Paulo, Brazil).

 The specimens were randomly distributed into 6 groups (n = 10) according to the type of endodontic sealer and universal adhesive used. The application and removal of endodontic sealers were performed according to the protocol described previously.

 AH-SU (AH Plus + Scotchbond Universal): After removing the epoxy resin-based sealer (AH Plus), the universal adhesive (Scotchbond Universal; 3M ESPE, St Paul, MN, USA) was actively applied for 20 seconds using a microbrush (KG Sorensen, Sao Paulo, SP, Brazil), gently air-dried, and light-cured for 20 seconds with an LED light-curing unit (Valo; Ultradent, South Jordan, UT, USA), at an irradiance of 1.000 mW/cm^2^.

 ES-SU (EndoSequence BC Sealer + Scotchbond Universal): Similar to the AH-SU group but using EndoSequence BC Sealer.

 MTA-SU (MTA Fillapex + Scotchbond Universal): Same procedure as the AH-SU group, but using MTA Fillapex sealer.

 AH-OU (AH Plus + OptiBond All-In-One): Similar to the AH-SU group, but using a different universal adhesive (OptiBond All-in-One; Kerr, Brea, CA, USA).

 ES-OU (EndoSequence BC Sealer + OptiBond All-In-One): Similar to the ES-SU group, but using OptiBond All-in-One adhesive.

 MTA-OU (MTA Fillapex + OptiBond All-In-One): Similar to the MTA-SU group, but using OptiBond All-In-One adhesive.

 Subsequently, four composite resin cylinders were made on each specimen using a transparent polyethylene cylindrical matrix (Tygon tube R-3603; Saint-Gobain Plastics, Maime Lakes, FL, USA), with an internal diameter of 0.7 mm and a height of 1.0 mm. The cylinders were made with a nanohybrid composite resin (Filtek Z350; 3M ESPE, St Paul, MN, USA). Two cylinders were positioned on the mesial and two on the distal surface of the dentin and light-cured for 40 seconds. The specimens were stored at 37ºC and 95% relative humidity for 24 hours before testing. Each specimen was fixed inside a metallic matrix, and each composite resin cylinders was aligned perpendicular to a 500-kgf load cell. An orthodontic wire (0.2 mm in diameter) was positioned at the base of each cylinder and pulled at a crosshead speed of 0.5 mm/min using a universal testing machine (EMIC DL2000, São José dos Pinhais, PR, Brazil) until failure. Bond strength values (in MPa) were calculated by dividing the maximum displacement force (in N) by the bonding area (in mm^2^). The arithmetic mean of the four cylinders was recorded as the mean bond strength for each specimen.

 Failure modes were assessed under a stereomicroscope (SZX7; Olympus, SP, Brazil), at × 30 magnification and classified as follows:

1. Adhesive: at the dentin‒adhesive interface
2. Cohesive: between the composite resin and the adhesive
3. Mixed: when both types of adhesive failure were present^23^

###  Statistical Analysis

 The data were tested for normality and homogeneity of variances using the Shapiro-Wilk and Levene’s tests, respectively. Data on the persistence of residue were analyzed using Kruskal-Wallis and Dunn’s test. Data on open dentinal tubules were analyzed using one-way ANOVA and post hoc Tukey tests. Microshear bond strength data were analyzed using two-way ANOVA and post hoc Tukey tests. The failure modes were analyzed descriptively. Statistical analyses were performed using SPSS for Mac v.25.0 (IBM, Armonk, New York, USA) with a significance level of 5%.

## Results

###  Persistence of Residues

 AH demonstrated the greatest persistence of residues among the groups (*P <*0.05). CON showed no residues on the dentin surface (*P* < 0.05), and there was no significant difference between ES and MTA (*P >*0.05). Table 2 shows the median, maximum, and minimum values and first and third quartiles of the scores attributed to the persistence of residues on the dentin surface.

**Table 2:** Median, maximum (max), and minimum (min) values, first (1Q) and third (3Q) quartiles of the scores for persistence of endodontic sealer residues after cleaning with 95% ethanol

	**CON**	**AH**	**ES**	**MTA**
median	0^A^	2^C^	1^B^	1^B^
max-min	0 - 0	3 - 2	1 - 1	1 - 1
1Q	0	2	1	1
2Q	0	3	1	1

^A,B,C^Different letters on the same line demonstrate significant differences (*P* < 0.05). CON, negative control; AH, AH Plus; ES, Endoequence BC Sealer; MTA, MTA Fillapex.

###  Open Dentinal Tubules Count

**Figure 2:**
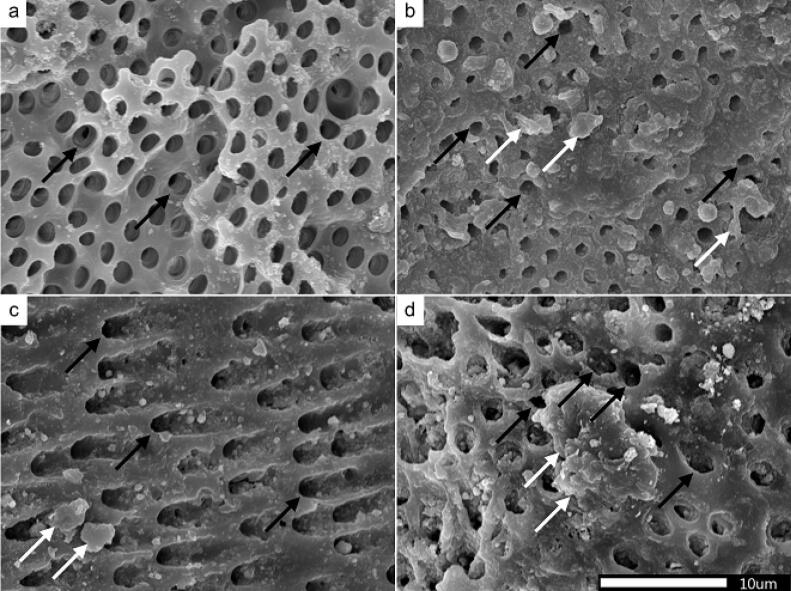
Representative images of the groups after cleaning with 95% ethanol. (a) CON, (b) AH, (c) Endosequence BC Sealer, and (d) MTA. White arrows show the persistence of sealer residues on the surface. Black arrows show available dentinal tubules on the surface

Table 3 shows the arithmetic mean and standard deviation of the number of open tubules on the dentin surface depending on the chemical composition of the endodontic sealer. AH and CON demonstrated the lowest and highest incidence of open dentinal tubules on the dentin surface (*P <*0.05), respectively. There was no significant difference between ES and MTA (*P >*0.05). Figure 2 shows the scanning electron microscope images of persistence of sealer residues and available dentinal tubules on the surfaces.

**Table 3:** Means and standard deviations (SD) of the count of open dentinal tubules on the dentin surface, after cleaning with 95% ethanol, according to the different endodontic sealers evaluated

	**CON**	**AH**	**ES**	**MTA**
Mean	144.10^A^	58.00^C^	94.80^B^	96.00^B^
SD	15.82	9.60	8.72	5.63

^A,B,C^Different letters on the same line demonstrate significant differences (*P* < 0.05). CON, negative control; AH, AH Plus; ES, Endoequence BC Sealer; MTA, MTA Fillapex.

###  Microshear Bond Strength Test and Failure Modes

Table 4 presents microshear bond strength values. ES-SU, MTA-SU, ES-OU, and MTA-OU groups demonstrated similar bond strength values (*P >*0.05), which were higher than those of the AH Plus groups, regardless of the universal adhesive system used (*P <*0.05).

**Table 4:** Means and standard deviations (SD) of microshear bond strength values, according to the endodontic sealer and universal adhesive used

	**AH-SU**	**ES-SU**	**MTA-SU**	**AH-OU**	**ES-OU**	**MTA-OU**
Mean	19.39^B^	24.26^A^	22.17^A^	19.15^B^	24.72^A^	22.96^A^
SD	15.24	12.83	15.45	17.53	18.06	32.58

^A,B^Different letters demonstrate significant differences (*P* < 0.05). AH-SU, AH Plus + Scotchbond Universal; ES-SU, Endosequence BC Sealer + Scotchbond Universal; MTA-SU, MTA Fillapex + Scotchbond Universal; AH-OU, AH Plus + OptiBond All-In-One; ES-OU, Endosequence BC Sealer + OptiBond All-In-One; MTA-OU, MTA Fillapex + OptiBond All-In-One.

**Figure 3:**
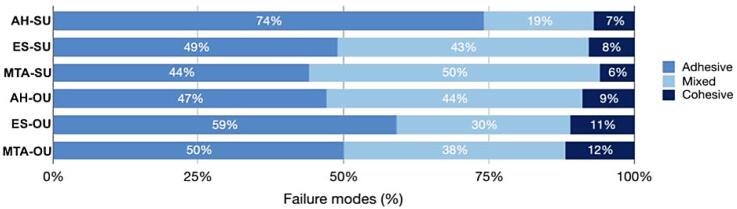
Incidence (%) of failure modes based on the endodontic sealer and adhesive system used

 The adhesive failure mode was the most frequent among the groups analyzed, except for MTA-SU, which showed a higher incidence of mixed failures. Figure 3 shows the failure mode incidence rates.

## Discussion

 Endodontic sealer residues on the adhesive interface with universal adhesives affect the longevity and performance of dental restorations. This study evaluated the effects of three endodontic sealers on the bond strength using two different universal adhesive systems. The findings demonstrated that the chemical composition of the sealer significantly affected both the persistence of residues and the adhesive performance of restorative systems. Residues of epoxy resin-based sealer on the dentin surface resulted in the lowest bond strength at the adhesion interface with coronal dentin, regardless of the universal adhesive used. Therefore, the null hypotheses were rejected.

 For effective dentin sealing, the dentin surface in the pulp chamber must be free of debris.^20^ Depending on the chemical composition of the endodontic sealer used, a specific cleaning solution may be more suitable than another. The sealer and the cleaning agent should exhibit similar polarity,^4,24^ in accordance with the principle that “like dissolves like,” where polar substances dissolve polar, and nonpolar substances dissolve nonpolar.

 Regarding the persistence of residues, AH Plus showed the highest scores, indicating greater retention on the dentin surface compared to the other groups (*P <*0.05). This finding is consistent with previous studies^2,20,25^ reporting that epoxy resin-based sealers adhere strongly to dentin due to their hydrophobic and cross-linked polymeric structure, which makes them less susceptible to removal by polar solvents such as ethanol.^20,26^ Calcium silicate-based sealers, being considered hydrophilic,^12^ responded more favorably to cleaning with ethanol (“polar substances dissolve in polar solvents”), resulting in less than 25% of residues covering the dentin surface. The evaluation of open dentinal tubules corroborates these findings. The highest number of open tubules was observed in the control group, while AH Plus demonstrated the lowest. No differences were observed between EndoSequence BC Sealer and MTA Fillapex.

 In clinical practice, the adhesive interface is subjected to a range of stresses, including mechanical forces from chewing, temperature fluctuations from hot and cold foods, and constant exposure to saliva. These factors can affect the long-term stability of the adhesive bond, particularly in the presence of residual sealer components.^10,22,27^ Thus, bond strength was also evaluated to analyze the effects of the cleaning protocol in association with different endodontic sealers. The results regarding the cleaning protocols and the performance of the bonding agents showed some similarities.

 The findings of this study demonstrated that epoxy resin-based sealer (AH) exhibited the highest persistence of residues on dentin surfaces, which was directly associated with the lowest bond strength values observed in both universal adhesive systems. Such residual deposits may hinder the infiltration of adhesive monomers into the dentinal tubules and compromise the formation of a stable hybrid layer, thereby weakening the adhesive‒dentin bond.^10,21^

 In contrast, the groups treated with ES and MTA sealers exhibited higher bond strengths than the AH groups. Universal adhesives contain functional monomers, such as 10-MDP (Scotchbond Universal) and GPDM (OptiBond All-in-one), which can chemically interact with calcium ions in hydroxyapatite, thereby forming stable ionic bonds with dentin.^13,14,28^ Although residual sealer was still observed on the dentin surface, the calcium released by calcium silicate-based sealers may have contributed to additional chemical interactions with the functional monomers of the adhesives.^29^ This phenomenon suggests that, rather than compromising adhesion, the residual bioceramic sealer could have acted as a source of calcium ions, enhancing bond strength, contrasting with the behavior of epoxy resin-based sealers, whose residues tend to form a physical barrier that hinders adhesive penetration.

 Although the adhesive systems have different chemical compositions and, consequently, may exhibit distinct performance regarding ionic bonding with hydroxyapatite,^13,14^ no statistically significant differences in bond strength were observed between them. Mixed failures were more frequently observed in the MTA-SU group, suggesting a more balanced contribution of both adhesive and cohesive mechanisms, while adhesive failures predominated in the other groups (Figure 3).

 The study highlights the importance of thorough cleaning protocols to minimize the persistence of residues and maximize adhesive bond strength. While 95% ethanol was used as the cleaning agent in this study, alternative agents or multi-step cleaning protocols could potentially offer superior results by more effectively removing sealer residues.^21,23^ Future research could investigate the efficacy of various cleaning agents, including solvent combinations, chelating agents, and ultrasonic cleaning, to determine the most effective methods for preparing dentin surfaces for adhesive bonding after endodontic treatment.^30^

## Conclusion

 Endodontic sealers containing calcium silicate, such as EndoSequence BC Sealer and MTA Fillapex, demonstrated lower persistent residues on the dentin surface following cleaning with 95% ethanol. These materials provided more favorable conditions for the adhesive interface with universal adhesives, regardless of their chemical composition.

## Competing Interests

 The authors declare that they have no competing interests.

## Ethical Approval

 In accordance with Normative Resolution No. 30/2016 of the Brazilian National Council for the Control of Animal Experimentation (CONCEA), ethical approval by an Animal or Human Research Ethics Committee was not required.
